# Determining non-cigarette tobacco, alcohol, and substance use typologies across menthol and non-menthol smokers using latent class analysis

**DOI:** 10.1186/s12971-017-0111-5

**Published:** 2017-01-17

**Authors:** Amy Cohn, Amanda Johnson, Jennifer Pearson, Shyanika Rose, Sarah Ehlke, Ollie Ganz, Raymond Niaura

**Affiliations:** 1 Schroeder Institute for Tobacco Research and Policy Studies at Truth Initiative, 900 G Street, NW, 4th Floor, Washington, DC USA; 2 Department of Oncology, Georgetown University Medical Center, 3970 Reservoir Road, NW, Washington, DC USA; 3 Department of Health, Behavior, and Society, Johns Hopkins Bloomberg School of Public Health, 624 N. Broadway Street, Baltimore, MD USA

**Keywords:** Cigarettes, Menthol, Alcohol, Drug use, Latent class analysis, Race, Cigarillos

## Abstract

**Background:**

Substance use and mental health are robustly associated with smoking and poor cessation outcomes, but not often examined in combination with menthol cigarette smoking, which is also associated with lower quit rates. This study identified classes of Black and White menthol and non-menthol cigarette smokers based on demographics, alcohol, drug, and other tobacco use behaviors.

**Methods:**

Using screening data from two studies, latent class analysis (LCA) was conducted to classify *n* = 1177 menthol and non-menthol cigarette smokers on demographic characteristics, heavy smoking, alcohol and drug use, desire to quit smoking, other tobacco product use, and use of psychotropic medication.

**Results:**

Three latent classes were identified that differentiated smokers on substance use, menthol cigarette smoking, and other tobacco use behavior. One class consisted primarily of young adults who used a wide array of other tobacco products, reported the highest prevalence of other drug use, and showed the lowest desire to quit smoking cigarettes in the next 6-months. Class 2 comprised primarily of Black male menthol smokers, all of whom used cigarillos in addition to cigarettes, and who displayed moderate drug use. The third class was categorized as primarily older cigarette smokers, who engaged in very little other tobacco use or drug use, but who were most likely to self-report being prescribed psychotropic medication.

**Conclusions:**

LCA allowed for the identification of distinct classes of smokers based on factors related to poor cessation outcomes, including menthol use, that have not previously been examined in combination. Interventions should target specific groups of smokers, rather than take a “one size fits all” approach.

## Background

While adult smoking prevalence has declined in recent years, prevalence rates remain high in certain vulnerable groups, including those with co-morbid alcohol, substance use, and mental health problems, and among menthol cigarette smokers [1–3]. Substance and alcohol use, mental illness, and menthol cigarette smoking are correlated with greater nicotine dependence [4–7], poly-tobacco use, increased risk of smoking relapse, and poor smoking cessation outcomes [1, 8–12]. Targeting smokers with mental illness is a high priority for both research and treatment.

Mental health, substance use, and menthol tobacco use could operate together to amplify negative tobacco-related outcomes, such as lower desire to quit smoking, poor smoking cessation success, and greater nicotine dependence. According to positive and negative reinforcement models of addiction, individuals with mental health and substance use problems may smoke cigarettes to relieve negative effects associated with mood instability or to heighten the experience of alcohol or other drug use through the stimulating properties of nicotine [13]. The cooling and soothing sensation of menthol cigarette smoking, which masks the harshness of inhaled cigarette smoke [14, 15], increases the rewarding and sensory properties of menthol smoking beyond those of nicotine alone and may be particularly appealing to individuals with substance use or mental health problems. Both Hickman et al. 2014 and Young-Wolff and colleagues (2015) found an elevated prevalence of menthol cigarette smoking among individuals with psychological distress [16, 17]; while Cohn et al. 2016 found higher rates of depression and anxiety in a national sample of young adult menthol tobacco users, relative to non-menthol tobacco users [18]. Further, qualitative work suggests that menthol cigarettes may heighten drug-related high and help to sustain euphoric mood [19]. Taken together, the high incentive value of menthol cigarette smoking, coupled with the rewarding and stimulating properties of nicotine on mood [13, 20, 21], may make cigarette smoking particularly appealing to certain vulnerable groups [22–24]. If menthol increases the subjective effects of nicotine on mood and substance use, one might expect greater uptake of menthol cigarettes to coalesce among those with more severe substance use behavior and among those with propensity toward mental health problems.

The associations among mental health, menthol cigarette smoking, and smoking behavior are complex and differ among sub-groups of smokers. Kiviniemi et al. 2010 found that psychological distress was correlated with higher smoking prevalence and increased odds of poor cessation outcomes among White, but not Black smokers [25]. However, menthol cigarettes are disproportionately used by Black smokers [26], and research shows elevated rates of psychological distress among menthol cigarette smokers relative to non-menthol smokers [16, 17]. However, there are no studies of differences across Black and White menthol and non-menthol smokers on mental health factors. Additional research shows that menthol smoking leads to worse cessation outcomes among Black and non-White menthol smokers, relative to White smokers [27]. Further, while menthol smokers consume fewer cigarettes per day and are less likely to report drug or alcohol use compared to non-menthol smoker [7], they show equivalent or higher dependence severity compared to non-menthol smokers [6, 28–31]. Further, unlike smokers with mental health or substance use problems, menthol smokers report a high desire to quit smoking [7, 32].

One approach for disentangling the complex associations among inter-related factors is through sub-typing techniques such as Latent Class Analysis (LCA). LCA is helpful for identifying a small set of qualitatively unique classes based on pre-defined person-level factors [33, 34]. Rather than examining correlations among factors, latent classes within LCA represent actual behavior that may be superior to fixed classifications (i.e., correlations), as these are based on estimated response probabilities. Latent class proportions can also provide information on the prevalence of how certain behaviors cluster together. For example, because LCA identifies unobservable groups with a population or sub-population, interventions can be tailored to target the subgroups that have the greatest need and whom may benefit the most from intervention. Applying LCA to determine unique sub-groups of already high-risk smokers could further refine “for whom” and “under what circumstances” public health messages about the harms and consequences of tobacco use could be best deployed.

Previous studies have used LCA to classify smokers. Some of these include classifying cigarette smoking behavior in college students [35] and adults [36], characteristics of alternative tobacco use among cigarette smokers [37], latent transition through the stages of smoking behavior change [38, 39], nicotine withdrawal severity [40], associations of depression to nicotine dependence [41], and determining gateway associations between substances of abuse including cigarettes [42]. Overwhelmingly these studies show that smokers are substantially heterogeneous. For example, in a sample of college students, Sutfin et al. [35] revealed five distinct classes of smokers, primarily distinguished by the frequency and intensity of their cigarette use, alcohol and drug use, and on the social aspects of smoking. Using a national sample of adult current smokers, Timberlake et al. [37] also revealed five classes of smokers, sub-typed on nicotine dependence severity and the use of alternative tobacco products. Results showed that moderate but not heavy smokers were most frequently using alternative, non-cigarette tobacco products. Despite the plethora of research in this area, no studies to our knowledge have taken an LCA approach to examine sub-types of Black and White smokers based on alcohol, substance use, and menthol cigarette use, factors that are robustly associated with poor smoking outcomes. Most importantly, menthol use should be included with alcohol and drug use when examining risk-typologies related to cigarette smoking because of its association with poor cessation outcomes and high policy relevance, and because of the high proportion of menthol cigarette smoking among Black versus White smokers.

Building upon prior research, this study attempts to fill an important knowledge gap by identifying unique classes of Black and White menthol and non-menthol cigarette smokers as a function of alcohol, drug, non-cigarette tobacco use, and other smoking-related risk factors. Findings provide a fine-grained assessment of the groups of adult smokers who should be differentially targeted by interventions and public health communication campaigns, and could have implications for targeting the most at-risk groups in tobacco control policies.

## Methods

### Participants and procedure

Data were combined from the screening measures of two NIH funded studies (data collected from January 2014 to December 2015). The first study was a naturalistic assessment of the longitudinal smoking change outcomes of risky drinking adult smokers. The second was an administrative supplement to this project, designed to examine motivations for alcohol use and cigarette smoking in adult smokers with and without a history of risky drinking. Both studies included the same battery of questionnaires at baseline (described below). Eligibility for both studies was nearly identical and included: 1) 18–65 years old; 2) smoke > 10 cigarettes per day; and 3) desire to quit smoking in the next 6-months. Exclusion criteria were 1) suicidal, homicidal, or severe psychiatric disturbance; 2) substance dependence (excluding nicotine and caffeine); 3) current use of psychotropic medication; and 4) potential for severe alcohol withdrawal. Additionally, individuals eligible for the parent grant drank at risky levels [>2 drinks/day for men, > 1 for women; and > 14 drinks/week for men; > 7 drinks/week for women, according to guidelines of the National Institute on Alcohol Abuse and Alcoholism [43]] and were excluded if they were pregnant or planning to become pregnant in the next 6-months. For the administrative supplement, a sampling method was employed to recruit 50% risky drinkers and 50% non-risky drinkers (consume at least 1 drink/week in the last 30 days but less than risky drinking levels). Participants in the current analysis completed the screening for these studies, but did not have to meet eligibility criteria to be included in the current analysis and not all participants who took the screening measure were risky drinkers.

Participants were recruited via online and print advertisements (e.g., Craigslist, local newspaper, and flyers) that asked for smokers who were regular drinkers. A total of 1177 participants completed the screening questionnaires and were included in the analyses described below.

### Measures

Latent class indicators included current menthol cigarette smoking (yes/no), current binge drinking (yes/no), other drug use in the past 90-days (yes/no), and non-cigarette tobacco use. Binge drinking was defined as consuming at least four drinks per episode for women and five drinks per episode for men “in a typical week” [44]. Current use of non-cigarette tobacco products was assessed by asking participants “Do you use any other tobacco products other than cigarettes” with response options for e-cigarettes, cigars, little cigar/cigarillos, hookah, chew, and “other.” Due to low prevalence rates, chew (3.8%) and “other” tobacco use (1.95%) were not included as indicators in the LCA.

Correlates of latent classes included age, gender, race, education, light (≤10 cigarettes per day) versus heavy (>10 cigarettes per day) smoking [45, 46], alcohol use (quantity and frequency in a typical week), prescribed medication for mental health problems (yes/no) as a 1-item proxy for mental health problems [47], use of chew and “other” non-cigarette tobacco products, number of non-cigarette tobacco products, and desire to quit or reduce smoking in the next 6-months (yes/no). Use of psychotropic medication was self-reported; respondents were asked whether they were “currently prescribed any medication for mental health treatment.”

### Data analysis

Latent class analyses were conducted in 2016 using Mplus 7.4. Variables were selected based on consensus agreement among all study authors and the literature. The optimal number of classes was determined by running models with a successive number of classes from one to five and comparing model fit indices. The optimal model was selected with the number of classes that minimized Bayesian Information Criterion (BIC), based on evidence showing that BIC outperformed other model fit indices in a simulation study [48]. Chi-square and t-tests were employed to identify differences in characteristics across latent class assignment with significance at *p* < 0.05. Finally, bivariate multinomial logistic regression models were employed to identify significant factors associated with class membership (*p* < 0.05).

## Results

### Latent class analysis

Model fit indices indicated best fit for a 3-class model (see Table 1). This model minimized the BIC, retained odds of correct classification greater than five across all classes, had entropy greater than 0.85, and was interpretable. Missingness on variables included in the LCA did not exceed 2%. Chi-square tests for missing completely at random (MCAR) under the unrestricted latent class indicator models for each model were examined and shown to be non-significant (*p* > 0.05).

**Table 1:** Optimal number of latent classes: assessment statistics

Model fit							
Latent classes	Number of free parameters	LL	BIC	Sample size adjusted BIC	LMR p-value	VLMR LRT p-value	Entropy
1	7	−4353.9	8757.3	8735.1	-	-	-
2	15	−4024.9	8155.9	8108.2	<0.0001	<0.0001	0.80
3	23	−3989.1	8140.9	8067.8	<0.0001	<0.0001	0.91
4	31	−3977.9	8175.0	8076.5	0.17	0.18	0.87
5	39	−3967.9	8211.6	8087.7	0.43	0.44	0.83
Odds of correct classification							
	Class 1	Class 2	Class 3	Class 4	Class 5		
1	∞						
2	12.5	22.8					
3	12.0	8.1	54.6				
4	3.0	8.6	4.1	40.7			
5	3.2	4.1	11.2	4.0	24.0		

Note. *LL* log likelihood, *BIC* Bayesian information criteria, *LMR* Lo-Mendell-Rubin, *VLMR* Vuong-Lo-Mendell-Rubin, *LRT* likelihood ratio test for *k* (H0) versus *k*-1 classes. Odds of correct classification (OCC) > 5 indicates a model with good latent class separation (Collins & Lanza, p. 74); OCC = ∞ indicates perfect classification

Table 2 displays the characteristics of the full sample and of each latent class. Class 1, the “Poly-Substance Users,” comprised 14% of the sample, was primarily young adults (Mean (M) age: 27.6; Standard Deviation (SD) = 0.6), and contained a slightly greater proportion of men than women. This class was highly educated, with 55.6% having completed some college and 24.7% having received a college diploma or greater. Nearly 60% were menthol smokers and 39.6% had used other drugs in the past 90 days. This class consumed an average of 3.0 (SD = 0.1) non-cigarette tobacco products, with hookah (88.3%), e-cigarettes (79.6%) and cigars (64.8%) being the most highly endorsed non-cigarette tobacco products. This class was the least likely to smoke heavily, relative to any other class (*p* < .002). While none of the groups varied significantly with respect to using alcohol, this class showed the highest prevalence of binge drinking behavior (57.4%). A significantly lower proportion of individuals in this class endorsed a desire to quit smoking compared to the other two classes (*p* < .017).

**Table 2:** Characteristics of the three latent classes (unweighted column %)^a^

	Poly-Substance Users	Menthol/Cigarillos Users	Primary Cigarette Users	Full sample	
	*n* = 162 (14%)	*n* = 170 (14%)	*n* = 845 (72%)	*n* = 1,177	*P* value
	%	%	%	%	
Age					
Mean (SE)	27.6 (0.6)	37.0 (1.0)	39.9 (0.5)	37.8 (0.4)	<0.0001
18–24	40.1	18.2	14.6	18.6	<0.0001
25–34	43.8	32.3	27.0	30.1	
35–45	13.6	21.8	18.7	18.4	
46+	2.5	27.6	39.8	32.9	
Gender					<0.0001
Female	40.1	29.4	49.4	45.2	
Male	59.9	70.6	50.6	54.8	
Race					<0.0001
White	38.3	7.6	35.5	31.9	
Black	61.7	92.3	64.5	68.1	
Education					<0.0001
Less than high school	3.7	10.0	8.1	7.7	
High school diploma	10.5	32.3	22.6	22.4	
GED	5.6	11.2	7.7	7.9	
Some college	55.6	32.3	39.3	40.6	
College diploma or greater	24.7	14.1	22.3	21.4	
Menthol use					<0.0001
Yes	59.3	88.2	62.1	65.5	
Heavy Smoking (≥10 cigs per day)					0.002
Yes	66.7	78.8	80.6	78.4	
Alcohol					
Binge drinking	57.4	51.2	52.4	52.9	0.449
# days used in typical week - Mean (SE)	4.2 (0.1)	4.4 (0.2)	4.3 (0.1)	4.3 (0.1)	0.430
# drinks per episode - Mean (SE)	5.4 (0.4)	6.4 (0.5)	5.5 (0.2)	5.6 (0.2)	0.089
Other drug use					<0.0001
Use in past 90 days	39.6	35.7	18.8	24.1	
Current tobacco use					
Cigars	64.8	19.4	3.2	14.0	<0.0001
E-cigarettes	79.6	5.9	5.6	15.8	<0.0001
Cigarillos	67.3	100.0	0.0	23.7	<0.0001
Chew	16.1	6.5	1.0	3.8	<0.0001
Hookah	88.3	12.4	3.2	16.2	<0.0001
Other tobacco	9.9	1.8	0.5	2.0	<0.0001
# tobacco products used					
Mean (SE)	3.0 (0.1)	1.7 (0.1)	0.6 (0.0)	1.1 (0.0)	<0.0001
Desire to quit in next 6 months					0.017
No	12.3	4.3	6.5	7.0	
Yes	87.7	95.7	93.4	93.0	
Mental health					0.081
Prescribed medication for mental health problem	11.1	6.5	12.5	11.5	

Note. *P*-values represent differences across the three classes. Chi-square tests were used for categorical variables and ANOVA F-tests were used for continuous variables

^a^ Italicized covariates are included as indicators in the LCA model

In Class 2 (14% of the sample; the “Menthol/Cigarillo Smokers”), 88% of individuals used menthol cigarettes as their preferred brand and 100% reported cigarillo use. The proportion of members in this class using menthol cigarettes as their preferred cigarette was significantly higher relative to the other two classes (*p* < .001). This class was primarily older young adults (M age = 37; SD = 1.0) and had the highest prevalence of Black and male respondents (all *p*’s < .001). Educational attainment was also lower in this class relative to the two other groups (*p* < .001), with most attaining up to a high school diploma or GED. Members within this class consumed the most drinks per episode (M = 6.4; SD = 0.5) relative to the other two classes, although the effect was marginally significant (*p* < .089) and 35.7% reported other drug use in the past 90-days. In addition to cigarillo use, use of non-cigarette tobacco products was prevalent in this class, with 19.4% reporting cigar use, 12.4% reporting hookah smoking, 6.5% reporting use of chew, and 5.9% reporting e-cigarette use. Just over three-quarters of this class consumed 10 or more cigarettes per day. This class contained the highest proportion of respondents wanting to quit smoking in the next 6 months (95.7%; *p* < .017)), and the lowest proportion reporting being prescribed medication for a mental health problem (6.5%), a proportion that was marginally different from the other two classes (*p* = .081).

Class 3 (the “Primary Cigarette Smokers”), comprised nearly 75% of the sample and had the highest mean age (M age = 39.9; SD = 0.5) and educational attainment, with 61.6% achieving some college education or higher. This class was the most likely to self-report heavy smoking (80%), being prescribed medication for a mental health problem (12.5%), and had the lowest prevalence of other drug use (18.8%) and non-cigarette tobacco use across the two other classes (*p* < .0001). E-cigarettes were the most popular non-cigarette tobacco product used (5.6%). Desire to quit smoking was also high in this class, with 93% reporting a desire to quit smoking in the next 6 months.

We conducted a sensitivity analysis of the LCA models to determine whether menthol use, drug, and alcohol use were driving class membership, above and beyond non-cigarette tobacco use. For this, LCA models were re-analyzed using only the four current tobacco product use variables (cigars, e-cigarettes, cigarillos, hookah) as class indicators. Results showed a 2-class model produced the best-fitting model (vs 3–5 models), suggesting it was more informative to include menthol use, binge drinking, and other drug use to identify typologies across menthol and non-menthol smokers. The 2-class model provided limited information on possible typologies, restricting the sample to two classes of either users (Class 1) or non-users (class 2) of non-cigarette tobacco products.

### Multinomial logistic regression models of characteristics of class membership

Results from bivariate multinomial logistic regressions of correlates of latent class assignment confirmed that the Primary Cigarette Smokers were significantly older than both the Poly-Substance Users and Menthol/Cigarillo Users. Compared to Primary Cigarette Smokers, membership in the Poly-Substance Users class was more likely to be male (OR = 1.46; CI: 1.04–2.05), three times more likely to be college educated (OR = 3.07, CI: 1.29–7.31), and twice as likely to use drugs in the past 90-days (OR = 2.82; CI: 1.97–4.06). Poly-Substance Users also had much higher odds of using a wide variety of non-cigarette tobacco products (OR = 76.48; CI: 45.72–127.95) compared to Primary Cigarette Smokers, even though they were significantly less likely to be heavy smokers (OR = 0.47; CI: 0.33–0.69). Poly-Substance Users were also significantly less interested in quitting smoking or reducing their cigarette intake in the next 6 months compared to Primary Cigarette Smokers (OR = 0.50; CI: 0.28–0.90).

Relative to Primary Cigarette Smokers, the Menthol/Cigarillo Smokers were twice as likely to be male (OR = 2.34; CI: 1.64–3.35), six times more likely to be Black (OR = 6.65; CI: 3.71–11.91), and four times more likely to be menthol smokers (OR = 4.57; CI: 2.87–7.44). Further, the odds of drug use in the past 90 days (OR = 2.39; CI: 1.67–3.43), and the odds of using a variety of non-cigarette tobacco products (OR = 18.52; CI: 12.18–28.14) was significantly higher among the Menthol/Cigarillo Smokers compared to the Primary Cigarette Smokers. Membership in the Menthol/Cigarillo class was significantly and inversely associated with self-reported prescription for psychotropic medication (OR = 0.49; CI: 0.26–0.93). There were no differences between the Primary Cigarette Smokers and the Menthol/Cigarillo Smokers with respect to heavy smoking (OR = 0.91; CI: 0.60–1.38) or desire to quit or reduce smoking in the next 6 months (OR = 1.56; CI: 0.69–3.51).

## Discussion

This study examined multiple typologies of tobacco-related risk in adult smokers based on non-cigarette tobacco use, menthol cigarette smoking, and alcohol and drug use. Our results found three distinct classes of individuals who differed with respect to race, gender, poly-tobacco use, drug use, menthol cigarette use, and desire to quit smoking. A Menthol/Cigarillo group was identified that was four times more likely to smoke menthol cigarettes compared to the Primary Cigarette Smokers, and significantly more likely to be male and Black compared to this group. Older smokers in the Primary Cigarette Smokers class showed fewer substance use and tobacco-related comorbidities, were also most likely to want to quit smoking in the near future, but seemed to have some mental health vulnerability as indicated by their reports of being currently prescribed psychotropic medication. Lastly, younger smokers were identified in a Poly-Substance Users class. These individuals were highly educated, non-heavy smokers who used a wide array of tobacco products, but were also the least likely to want to quit smoking. While prior research shows that greater educational attainment is associated with interest in quitting and quit attempts, this work has been explored primarily among older adult samples [49]. Poly-substance users in the current analysis were young adult users. This is a group that has been shown to be less likely to be motivated to quit using tobacco or to engage in tobacco cessation compared to older age groups [50]. Poly-Substance Users and Primary Cigarette Smokers were equally likely to report menthol smoking. Ultimately, menthol use did not seem to be distinctive among two of the three classes, rather classes were distinguished by non-cigarette tobacco use and illicit drug use. These findings could be due to the high rates of menthol use in the sample overall. Lastly, sensitivity analyses showed that including menthol, alcohol, and drug use in addition to non-cigarette tobacco use were important indicators in determining typologies across menthol and non-menthol smokers.

The identified latent classes may have important implications for whom to target in public health anti-tobacco campaigns; suggesting that different messages be deployed for young versus older smokers, and for Black menthol smokers versus other groups of smokers. Specifically, communications surrounding poly-tobacco use may be effective for younger audiences, while messages about the harms associated with menthol smoking should be directed toward Black males, especially those who consume LCCs. Poly-substance use must also be addressed among today’s current smokers, particularly younger smokers, as is consistent with several studies of young adult dual and poly-tobacco use [51–55]. Interestingly, relative to the other two groups, the Poly-Substance Users class were lighter smokers, but had the highest prevalence of non-cigarette tobacco use and the lowest desire to quit or change smoking. This may suggest a willingness to try new products or a vulnerability to engage in a variety of health-risk behaviors [56], which may impede efforts toward positive behavior change. Perhaps, this class could be a target for health promotion campaigns that focus on enhancing motivation to quit and on addressing the health impact of poly-tobacco use. Because alcohol consumption across the three groups was high, this also highlights the need to thoroughly address alcohol use in nearly all tobacco dependence treatment intervention and prevention programs, regardless of age or race.

Application of LCA in this study also has several scientific and policy-level implications. First, LCA models allow us to understand the complex patterns and associations among tobacco use, mental health, substance, and race in greater detail than has been previously pursued. While there have been efforts to classify smokers as a function of alcohol, substance use and non-cigarette tobacco use in other LCA studies, none to our knowledge have included menthol cigarette smoking as a key indicator of class membership. It is important to focus on menthol tobacco use in the context of alcohol and other drug use, as each are independently associated with poor smoking cessation outcomes, but only a few published studies have examined how these risk factors operate together [7, 18, 57]. It is possible that, when combined together, substance use and mental health problems, poly-tobacco use, and menthol cigarette smoking may interfere with motivation to quit smoking. Further, with respect to menthol tobacco use in particular, most previous studies have conceptualized menthol smokers as a homogenous group and made comparisons to non-menthol smokers or have examined differences across Black and White menthol and non-menthol smokers [25, 27]. Our LCA approach allowed us to disentangle potential sub-groups of menthol smokers, not based only on race, but on other tobacco use correlates. Finally, by using LCA to delineate subgroups of smokers who have various levels of tobacco-related risk, it may be possible to understand how policy interventions may differentially impact subgroups of smokers. It is noteworthy that one unique class of menthol smokers emerged, all of whom consumed little cigars and cigarillos (LCCs), while a second class of predominantly menthol smokers did not. With the rising costs of cigarettes, the Menthol/Cigarillo group may represent a sub-set of smokers who are extremely price sensitive and who may be substituting menthol cigarettes for cheaper menthol flavored LCCs. This has important implications if a ban on menthol cigarettes, but not other menthol tobacco products, was set in place. These individuals may switch from smoking menthol cigarettes to menthol flavored LCCs.

This study had several limitations. First, prior cessation experiences and nicotine dependence were not measured, limiting our ability to examine the extent to which class membership is associated with tobacco use persistence and problem severity. Further, absolute number of cigarettes consumed per day was not assessed; rather, we used a categorical variable capturing heavy (10 or more cigarettes per day) and light smoking, an approach consistent with other studies of smokers [46]. Second, because this was a secondary analysis of previously collected data, we did not have an instrument to measure mental illness with more detail. Our assessment of mental health status, as indicated by self-reported prescription of mental health medication, has been used in other work [47], but is not a diagnostic tool. Given that the majority of individuals with a mental health issue receive no treatment [58, 59], this indicator likely underestimates the prevalence of mental illness in the current sample. Third, because study advertisements asked for smokers who are regular drinkers, this might explain why alcohol use was high across all the classes. It is important to note that our sample did show variation in alcohol use, ranging from no drinks up to 25 drinks per drinking episode, suggesting that not all respondents in our study were heavy drinkers. Further, the population prevalence of non-drinking smokers is relatively low [60]. Finally, the sample was comprised of primarily Black smokers. This is likely a reflection of the urban community from which participants were recruited, which has a high prevalence of African-American residents. Other studies have used primarily White samples [57], which also bear the same generalizability issue but in the opposing manner, or do not report the racial/ethnic breakdown of their full sample [3, 61–63].

## Conclusions

Our findings indicate a need for further research to substantiate classification of smokers according to menthol cigarette smoking, substance use and mental health, and non-cigarette tobacco use. Despite the proliferation of anti-tobacco campaigns, particularly aimed at youth and young adults, non-cigarette tobacco use in some age groups has been on the rise, indicating a need for more effective approaches to reach those who are not amenable to change. Sub-dividing groups can improve tailored interventions to those most at need, based on different psychosocial and behavior profiles and tobacco use risk factors.

## Abbreviations

BIC: Bayesian Information Criterion
LCA: Latent class analysis
LCCs: Little cigars and cigarillos
M: Mean
MCAR: Missing completely at random
SD: Standard deviation
